# Duties of the Medical Profession in View of War

**Published:** 1917-05

**Authors:** 


### Duties of the Medical Profession in View of War.

The medical profession, through its intimate contact with the people can exert its influence in a variety of ways.

It should encourage the naturalization of foreigners. Aliens born in enemy countries cannot now take steps to secure citizenship, but beyond registration and exclusion from the vicinity of military posts and points of military vulnerability, it is not now proposed to subject them to inconvenience, provided they avoid hostile speech and acts. As any civilian manifesting hostility is more or less in the category of spies, those who cannot conscientiously avoid such manifestations should be urged for their own protection, if for no other reason, to surrender themselves as prisoners of war. Unfortunately, this applies directly to a small part of the profession.

It is the obvious duty of loyal citizens and residents to give information of spies and plotters, but common sense should be exercised to avoid an injury to well disposed aliens as well as to avoid overwhelming the secret service with unfounded suspicions.

The ultimate military power of any nation depends, as never before, and especially to this country under existing circumstances—which reduce its actual military activities in the scale of immediate importance—upon general industrial efficiency and economy. For the average citizen, especially the one who has reached a place of fair usefulness, and who has dependents who would, by his absence on military service, ultimately require public assistance, the most practical patriotism is to hold his present job, to increase his efficiency, and only slightly if at all to increase his charges for his service to others.

Mere entrance into war has cost every average man, woman and child $70 in direct liability. This means about $1,000 for every head of a family of moderate prosperity, as the average physician. We can easily stand this expense. The factor that strikes hardest at the spirit, patriotism and resistance of a people, is not the direct tax and the national debt assumed, but the indirect tax in increased price of necessities, which in our country, with its enormous productive power, will be due almost entirely to unrestrained speculation and exploitation. The country has already appointed a food director, and the people should insist that his function should be immediately and efficiently performed. Deliberate wholesale destruction or sequestration of food, as has been frequently practiced to maintain artificially high prices, should now be recognized as destruction of life, and offenders should be dealt with in accordance with general principles of martial law. The same applies, only less strictly, to gasoline, leather, wool, cotton, coal, building material and every other necessity of life.

One of the most practical and immediately necessary patriotic duties is to restrain unwise expressions of patriotism. Expressions of race hatred, prejudice against distinguished individuals of enemy countries, against the language, science and art of such countries, advocacy of cruel measures of war and the like, are cheap and foolish ways of manifesting loyalty, and do almost as much harm as expressions of disloyalty. It should be remembered that at least a fifth of the population of this country, and a larger proportion of the science and art of medicine, are ultimately derived from what are now enemy countries. In no way can we alienate sympathies for our own country and lead to actual danger, more than by puerile expressions of prejudice.

Another very practical form of patriotism is to recognize clearly that the protection of our country must depend mainly upon its regularly organized forces. With the exception of offers of unusual magnitude and of unusual forms of service and commodities, not easily obtained, the burden of war can be best borne by general, equable distribution, as directed by the government. The financial strength and available resources of the country can easily be dissipated in wasteful forms of private and semi-private philanthropy, which duplicate and confuse the functions of government. Even the time and strength of officials may be severely taxed by unnecessary resolutions of commendation and offers of assistance.

Every man should, so far as practicable, estimate how he can best serve his country and how his country can best get into touch with him. It is a mistaken notion that the highest service we can render is to die for our country. Exactly the opposite is true. The brunt of the actual fighting must be borne by the young, not only for physical reasons, but because the man who weakens the economic strength of the country by leaving useful work and the care of dependents to be taken up by the people, does more harm than good. Similarly, the man who is competent to perform, either in direct military service or economically, functions of high order that cannot easily be filled by others, sacrifices his country in sacrificing himself. This applies especially to the medical profession and to students in various technical schools, including medical, who can better serve their country by completing their education than by enlisting prematurely.

Physicians, in particular, should recognize the various ways open to them, in and out of military and quasi-military service, should decide in accordance with common sense or in accordance with actual demands by the government, and should realize that it is not true patriotism to attempt to do more than one thing at a time.

The influence of physicians in their clientele may be equally valuable in directing patriotic aspirations and in checking unwise volunteering and, on the other hand, in rebuking those who seek to avoid an obvious duty, as for example in what has become too common, the attempt to hide from military service behind the skirts of a bride.

We are authorized to state that the Red Cross is recognized by the Medical Department of the Army as a worthy aid, but care should be taken to conduct its local activities in conformity with the spirit and technic of its organization, so that any unit can be promptly made available. Otherwise, medical preparedness should be on a personal or informally associated basis so as neither to conflict with the availability of individuals for more formal military service, nor to confuse and misdirect the normal medical energies of the country. For example, it is questionable whether a civil hospital should be organized on a military basis. It is extremely doubtful whether such hospitals will be needed by the army and navy, or whether they could be used without detracting from the established and equally necessary service to the civil population. A more practical plan would be for each hospital to estimate how many military patients and of what kinds, could be properly cared for, if necessary, without interfering with the regular work of the hospital. We question even whether the proper time has arrived for thrusting this offer upon a staff already greatly overburdened. It is scarcely conceivable that such services will be needed for several months, at least. In formulating mobile units for military service, certain questions should be asked. Absolute sincerity free from desire for notoriety and exclusiveness need not be mentioned as prime requisites, but the units as formulated should be actually in accordance with Red Cross standards, or should be left incomplete. Will the members of a unit be promptly available for mobilization as required, are they physically fit for the exigencies of active service, who will take their places in the necessary work which they will leave behind, how will they be trained for the special duties, including what the civilian scornfully but rather unjustly calls red tape, does membership in such a unit conflict with services already volunteered or due in some other way, or do such units draw upon material which should be available for regular service? This last point is especially important, and in regard to it we quote a statement from the Surgeon General's office: "The promise of a duty such as would satisfy the conscience of some, coupled with a guarantee that there will be none of the discomforts of field service, would undoubtedly appeal to a large number of persons in the medical profession as well as in other walks of life." This is immediately qualified, however, with a high compliment to the patriotism of the medical profession in general.

